# Qualitative outcomes of Clean Cut: implementation lessons from reducing surgical infections in Ethiopia

**DOI:** 10.1186/s12913-019-4383-8

**Published:** 2019-08-17

**Authors:** Aviva S. Mattingly, Nichole Starr, Senait Bitew, Jared A. Forrester, Tihitena Negussie, Sylvia Bereknyei Merrell, Thomas G. Weiser

**Affiliations:** 1 Stanford University School of Medicine, Palo Alto, CA USA; 2 Department of Surgery, University of California San Francisco, San Francisco, CA USA; 3 Lifebox Foundation, London, UK and Brooklyn, NY USA; 4 Department of Surgery, Division of General Surgery, Section of Trauma & Critical Care, Stanford University School of Medicine, Palo Alto, CA USA; 5 Department of Pediatric Surgery, Addis Ababa University, Addis Ababa, Ethiopia; 6 Department of Surgery, Stanford-Surgery Policy Improvement Research & Education Center (S-SPIRE), Palo Alto, CA USA

**Keywords:** Ethiopia, Quality improvement, Surgical infection, Resource limited

## Abstract

**Background:**

Clean Cut is a six month, multi-modal, adaptive intervention aimed at reducing surgical infections through improving six critical perioperative processes: 1) handwashing/skin preparation, 2) surgical gown/drape integrity, 3) antibiotic administration, 4) instrument sterility, 5) gauze counts, and 6) WHO Surgical Safety Checklist use. The aim of this study was to elucidate themes across Clean Cut implementation sites in Ethiopia to improve implementation at future hospitals.

**Methods:**

We conducted semi-structured interviews of 20 clinicians involved in Clean Cut at four hospitals. Participation was limited to Clean Cut team members and included surgeons, anesthetists, operating room (OR) nurses, ward nurses, OR managers, quality improvement personnel, and hospital administrators. Audio recordings were transcribed and coded using qualitative software. A codebook was inductively and iteratively derived between two researchers, tested for inter-rater reliability, and applied to all transcripts. We conducted thematic analysis to derive our final qualitative results.

**Results:**

The interviews revealed barriers and facilitators to the implementation of Clean Cut, as well as strategies for future implementation sites. Key barriers included material resource limitations, feelings of job burden, existing gaps in infection prevention education, and communication errors during data collection. Common facilitators included strong hospital leadership support, commitment to improved patient outcomes, and organized Clean Cut training sessions. Future strategies include resource assessments, creating a sense of responsibility among staff, targeted training sessions, and incorporating new standards into daily routine.

**Conclusions:**

The findings of this study highlight the importance of engaging hospital leadership, providers and staff in quality improvement programs, and understanding their work contexts. The identified barriers and facilitators will inform future initiatives in the field of perioperative infection prevention.

**Electronic supplementary material:**

The online version of this article (10.1186/s12913-019-4383-8) contains supplementary material, which is available to authorized users.

## Contributions to the literature

- Research has shown that quality improvement programs in low and middle income countries have unique challenges
- We undertook a qualitative review of a comprehensive surgical quality improvement program in Ethiopia, and identified key barriers and facilitators to successful implementation in the hospitals within which this was undertaken
- These findings help understand the considerations involved in implementing global surgery programs, and provide important themes for researchers to reference when establishing new programs in similar settings

## Background

In 2008, the WHO Surgical Safety Checklist (SSC) was introduced as part of a global initiative to improve surgical safety, and demonstrably reduced post-operative morbidity and mortality [1]. The SSC is now used in operating rooms worldwide, but its implementation and adoption has been variable [2]. This is part due to numerous barriers to compliance with elements embedded within the checklist, engrained hierarchy, entrenched attitudes and behaviors, and ineffective management. Challenges in compliance are not unique to the SSC, and exist around various hospital guidelines, including those regarding emergency procedure guidelines and protocols to reduce central line infections [3, 4].

Due to the difficulty in complying with items incorporated into the WHO Surgical Safety Checklist, a number of developers of the checklist founded Lifebox, an organization dedicated to improving surgical safety. Using the SSC as a framework, Lifebox has created a number of programs focused on improving anesthetic safety, reducing surgical infections, and improving teamwork. One of these programs is Clean Cut, an adaptive, multimodal quality improvement intervention that focuses on key perioperative infection prevention standards to reduce surgical infections (SSI) [5]. Currently deployed at eight hospitals in Ethiopia, Clean Cut involves a team of surgeons, nurses and anesthesia providers who drive perioperative procedural improvements and collect surgical outcomes data over the course of 6 months. Data collection focuses on compliance with six infection prevention standards that include: 1) skin and hand decontamination, 2) maintenance of the sterile field, including integrity of gowns and drapes, 3) antibiotic timing and selection, 4) sterility of instruments, 5) surgical gauze counts, and 6) use of the SSC; it also assesses surgical outcomes in the form of postoperative infections, deaths, length of stay, and reoperations.

Previous mixed-method and qualitative studies have analyzed checklist initiatives in both high and low resource settings. Implementation of the SSC varies from nationwide mandates (i.e. in the UK), to individualized implementation projects and partnerships similar to Lifebox interventions. Research shows that there is greater variation in the use of the SSC in low and middle income countries (LMICs) which may indicate increased barriers to use [6]. Previously described barriers include resource constraints, cultural barriers, staff resistance, lack of established data collection methods and negative effects of hierarchy [7, 8], indicating that checklist implementation requires a multifaceted approach specific to the local context. Contextual differences are essential to consider when implementing the SSC in any setting, but in LMICs there may be distinct barriers not readily apparent in high income countries.

Despite the aforementioned research, there is limited qualitative literature describing the process of implementing surgical safety projects globally. By understanding the unique challenges of quality improvement work in resource-constrained settings, perioperative teams will be better equipped to improve patient safety through hospital partnerships and training programs. The aim of this study is to elucidate common themes across hospitals implementing Clean Cut to better understand the barriers and facilitators of implementing this quality improvement program and provide insight for additional facilities within and beyond Ethiopia.

## Methods

### Setting

Clean Cut was piloted at Jimma University Specialized Hospital (JUSH) in Jimma, Ethiopia. It was subsequently implemented in four other facilities: Tikur Anbessa Specialized Hospital (TASH) and Menelik II Referral Hospital (MII) (both affiliated with Addis Ababa University) and St Peter’s Hospital in Addis Ababa, and Fitche Hospital in Oromia, Ethiopia. Each hospital provides surgical care and has a combined catchment population of 25 million people [9]. In particular, TASH is the largest referral hospital in the country. The program was implemented between August 2016 and August 2018. For this study we focused on interviewing participants at the latter 4 Clean Cut implementation hospitals. Two hospitals (St Peters and Fitche) were nominated by the Ministry of Health and the other two demonstrated strong engagement by surgeons and nurses; TASH is also the largest teaching hospital in the country and the clinical base of one of the authors (TN).

### Interviews

We conducted a qualitative study using semi-structured interviews over four weeks (July–August 2018) in Addis Ababa, Ethiopia. This study was approved the by the Stanford University and Addis Ababa University IRBs. Recruitment for interviews was purposefully targeted towards all Clean Cut team members directly involved in implementation to allow for meaningful programmatic feedback. We included surgeons, anesthetists, OR nurses, ward nurses, OR managers, quality improvement personnel, and hospital administrators; each was approached in person or by phone. At each Clean Cut site, at least 3 team members were also chosen to be dedicated data collectors (generally OR and ward nurses). When study interviewees were data collectors in addition to their other role, this was identified during the interview. Interviews were conducted in-person at the hospital site and in English (AM, NS), although translation was performed by one of the authors (NS) or the local facility clinical lead for participants with limited English skills. We aimed to recruit at least 15 participants based on literature suggesting 12 interviewees are typically needed to reach thematic saturation [10]. A previously agreed upon structured interview guide was used for each interview and modified based on participant’s role in the hospital. The interview guide was developed by several of the authors (AM, NS, SB, TW, JF) for this study to inform improvements in the Clean Cut program [11]. The interview guide was created based on feedback from piloting of the work at the first hospital site and observed barriers and facilitators in that setting (see Additional file 1: Clean Cut Qualitative Interview Guide). Each participant was interviewed once; there was no financial incentive included. Audio recordings were obtained following either written or verbal permission after informed consent on an encrypted device, de-identified, and transcribed using a professional transcription service. Each transcription was further reviewed and edited by the lead author (AM) for any transcription errors before uploading to the qualitative analysis software Dedoose (version 8.1.8 Los Angeles, CA: SocioCultural Research Consultants, LLC www.dedoose.com).

### Qualitative coding and analysis

A codebook was inductively and iteratively derived and applied to transcripts from all sites [12–15]. To create the codebook, one author (AM) first established three major domains based on constructs from the interview guide: 1) process measure changes, 2) barriers and facilitators, and 3) strategies for implementation. Based on these larger domains, the transcripts were inductively coded starting with one transcript from each hospital site to capture a diversity of ideas. Co-authors (TW, NS) further developed and approved the preliminary codebook [16]. The codebook was reapplied to previously coded transcripts. An inter-rater reliability test (IRR) was performed with an outside researcher unfamiliar with the project to gain additional feedback. After revisions of both codes and code definitions, the coding team (AM, NS) performed another IRR test before proceeding with an IRR goal of a minimum kappa of 0.7 [17]. After all transcripts were coded, the primary coding team met in person for a final agreement process and thematic analysis [18]. Extracted themes were then assessed to identify barriers and facilitators to implementation.

## Results

We interviewed 20 total participants across all 4 hospitals; interviews ranged from 25 to 60 min in length. Two individuals were affiliated with Lifebox but not regularly involved in Clean Cut implementation, and thus excluded from this analysis. Three of the four hospitals were located in Addis Ababa, Ethiopia, while the 4th (Fitche) was located in a rural region outside of the city. The participants occupied a variety of roles in the hospital (Table 1).

**Table 1:** Demographics of Participants by Hospital

Characteristics	Menelik II	Tikur Anbessa	St. Peter’s	Fitche
Total Participants^a^	4	6	6	4
Female	1	4	1	1
Role^a^				
Clinical Lead	0	1	1	1
Data Collector	3	2	3	3
OR Manager	1	0	1	0
Surgeon	1	3	1	1
Anesthetist	0	0	0	1
Nurse	3	2	3	2

^*a*^ *total participant numbers are less than the number of roles as participants may be listed as data collector or clinical lead in addition to their professional title*

The major barriers and facilitators to implementing Clean Cut fell into 5 main categories: i) material resources, ii) hospital administration, iii) hospital staff, iv) knowledge, beliefs, and education, and v) data collection (Table 2). Within each category a number of themes were identified that served as specific barriers and facilitators to implementation of the program.

**Table 2:** Barriers and Facilitators

	Barriers	Facilitators
Material Resources	● Availability of autoclaves, indicators, soap and alcohol hand rub ● Availability of paper and printer ● Budget restrictions	● Hospital investment in indicators, soap, and alcohol hand rub ● Access to biomedical engineering services
Hospital Leadership	● Lack of awareness	● Administration buy-In ● Purchasing power
Hospital Staff	● Job Burden ● Resistance to SSC use during emergencies ● Fear of punishment in the workplace and/or legal consequences	● Motivations based on improving patient outcomes ● Individual (financial) incentives ● Positive team dynamics ● 1:1 Conversations ● Personnel with prior QI experience
Knowledge, Beliefs & Education	● Belief that there is no problem of SSI in Ethiopia ● Misreported data on SSI ● Misunderstanding of sterility confirmation	● Orientation by Lifebox ● Presentations and conversations regarding perioperative evidence-based infection prevention practices
Data Collection	● Handwriting errors ● Confusion among staff ● Delayed payment ● Insufficient “manpower” ● Difficulty with patient follow-up	● Teamwork and division of responsibilities ● Creating a schedule for data collection

*SSC* surgical safety checklist, *SSI* surgical site infection

### Barriers

#### Material resources

The four study sites differed in the amount of material resources available for surgical procedures and data collection. There was difficulty in completing SSC tasks and Clean Cut process measures due to lack of surgical equipment. At one site there was no functioning steam autoclave, and at another site there was a lack of distilled water leading to suboptimal sterilization:

*“ … using an autoclave with the distilled water, it has a lot of benefits. But there is no distiller available. So you are going to use it with tap water, and it will be damaged...” (OR Manager 1)*

Similarly, indicators to confirm proper sterilization were not always available. One out of the four sites did not have any sterility indicators available at the beginning of Clean Cut implementation.

Sterile technique was also limited by holes in gowns and drapes. Even in cases where OR staff were trained to discard faulty gowns and/or drapes, there was a need to reuse these resources because there was no option for replacement:

*“The budget is not allowing us to have it, whenever we ask them. Otherwise that’s why a gown or drape, whenever it is broken, is not thrown away. It is remade and reused again. We are not in the luxury of throwing away those nonfunctional instruments or gowns or drapes.” (Surgeon 1)*

Improved hand washing practices were limited by the availability of alcohol rub at most hospital sites. Despite improvements in staff knowledge of proper hand decontamination, there were still shortages of alcohol hand rub limiting compliance.

Lastly, material resources were a barrier to data collection because paper supplies and computer and printer access limited the ability of data collectors to perform their role. In one case, the hospital staff in a rural setting needed to travel to the capital city monthly to gain computer access for data input. In that same setting, the only printer available was in the CEO’s office. In the larger urban setting, computer access was not a problem, but paper for printing data collection forms or the SSC was sometimes unavailable.

#### Hospital leadership & management

The barriers at the hospital leadership level were primarily due to a lack of understanding between the administration and Clean Cut members. At times the purpose and structure of the Clean Cut program were not clear to hospital administration. One participant noted that poor leadership was a generalized barrier in the hospital setting and not specific to Clean Cut:

*“There is a saying in Amharic [Ethiopian Semitic Language] ...When a dead fish stinks, it starts from the head... they [leadership] would not have a system for people to come and express their feeling, their thinking of how the system should operate. It was just somebody having an idea and telling them, “Do this. Do that.” A paternalistic way of leading the organization in general. That's not good. That's not going to bring changes.” (General Practitioner 1)*

When senior leadership was involved, including the CEO, there was still a challenge of ongoing management. Leaders could approve the purchasing of necessary resources, establish new procedures, and encourage adherence to new perioperative guidelines, however this was not always the case after the initial program meeting:

*“The difficulty is to adopt or to stick to the guidance. If you got feedback from [Clean Cut], then we are not applying it. Whether it's a financial reason or sometimes people from the administrative staff, they may not buy something early.” (Surgeon 2)*

#### Hospital staff culture & behavior

Among staff members who were directly involved in perioperative Clean Cut tasks, one barrier was the increasing burden on daily workload. Staff perceived that completing the SSC in the OR delayed their work:

*“When we do the time out or the sign-in, they see it as delaying their job or something. Because the anesthetists … just intubate the patient and they don't want the checklist to delay their work. And also the surgeons, they just want to start right away but now, it's changing.” (Data Collector/Nurse 1)*

In addition to perceived job burden, there was also the issue of whose responsibility it was to complete the SSC. Clean Cut team members undertaking data collection were compensated for this; paid data collection became associated with completion of the SSC at some sites. We found that other staff felt that they were not responsible for any part of Clean Cut, including completion of the SSC, if not paid. Some OR nurses outside the Clean Cut team perceived completion of the SSC as strictly the responsibility of Clean Cut team members rather than a standard part of safe OR practices.

*“Since the Clean Cut team got additional payment for the data collection from the Clean Cut project, then [the other OR nurses] expect us to fill this surgical safety checklist … They perceived as it is our job.” (Surgeon 2)*

One participant noted that a barrier to checklist completion had to do with emergency procedures. When preparing a patient for urgent or emergent cases, such as trauma operations or emergency Cesarean sections, the SSC was perceived as time-consuming and a low priority. Lastly, the accountability that came with completing Clean Cut data forms had negative consequences. Some individuals feared, *“If they didn’t complete everything to the checklist … if they didn’t count the gauze, then they were going to be punished”* (Data Collector/Nurse 2). Additionally, assigning an individual to the completion of the SSC resulted in fear of medicolegal consequences by some staff if the patient went on to have a complication.

#### Knowledge, beliefs, and education

Barriers related to knowledge and beliefs included poor familiarity with surgical best practice guidelines and misunderstanding of the Clean Cut intervention itself. For example, staff often had differing beliefs regarding hand washing practices and confirmation of surgical instrument sterility that did not align with standard guidelines. These gaps hindered practice changes, as participants noted: *“the resistance to change was the lack of knowing things” (Data Collector/Nurse 3).*

In addition to not knowing all of the standard procedural guidelines, participants also did not have accurate information of the level of surgical site infection in Ethiopia or at their specific hospital. Several participants reported seeing national data that the infection rate was as low as 1%, while Clean Cut monthly baseline data revealed much higher rates, ranging from 2.8 to 16.92% at individual facilities.

#### Data collection

There were several clear barriers to data collection. At all sites, data collection was done on paper forms, which lead to errors when handing off patients or inputting data into an online file. Second, all data collectors were compensated for their work during the study. At times payment was delayed, which created inconsistencies or breaks in data collection. Smaller sites reported personnel shortages, which exacerbated the extra burden of data collection. In some cases, staff worked 6–7 days of the week including holidays and weekends when there was no other person to cover. Lastly, the staff reported difficulty with patient follow up because many patients did not have phones, particularly in rural areas.

### Facilitators

#### Material resources

Investment in material resources facilitated improvements in both SSC and other Clean Cut process compliance. When a hospital invested in autoclave sterility indicators, soap and alcohol hand rub, as well as water distillers, it was able to improve practices around drape, gown and instrument sterility, and proper handwashing. With regards to the autoclaves, when there was biomedical engineering maintenance available, there were fewer reported issues with autoclave function.

#### Hospital leadership & management

Hospital administration engagement facilitated successful implementation. Buy-in from upper management encouraged staff to bring new ideas forward throughout program implementation. Participation by hospital administration also brought a sense of common purpose to the program.

*“In our hospital, we have very committed CEO. We have very committed medical director...The level of commitment you see in the higher officials, it's reflected on the workers down there. The level of commitment I see from ... senior surgeon, is what drives me to work harder every day.” (General Practitioner 1)*

When there was an open line of communication, the hospital administration’s purchasing power was leveraged to fix resource issues as they came up, including maintenance of the autoclave and purchasing sterility indicators. One CEO ensured a supply of soap and provided printer access for data collectors. Any practice changes requiring capital, ongoing expenditures, or restructuring workforce responsibilities were more successful if hospital administration was engaged in the program.

#### Hospital staff culture & behavior

We identified two major forms of incentives for hospital staff. Motivations based on patient outcomes were noted across all study sites, as all staff were inspired to reduce morbidity and mortality. Participants reported wanting to understand the sources of infection in their facility and how they could prevent patient infections, and thus patient outcomes.

*“ … almost everyone is a stakeholder, because it all comes down to the patient, and every one of us, we are working to improve patient outcome. [The] Checklist is everybody's responsibility.” (Surgeon 3)*

These motivations led to specific improvements in wound care precautions in the hospital setting and more complete discharge instructions. One hospital noted that the technique of wound care had been changed through the use of sterile gloves, cleansing the wound area more frequently and not leaving the wound open for extended periods. Upon discharge, communication increased with patients, *“They [nursing staff] will take signs of infections” and “they [nursing staff] will advise them [patients] to come early” (Data Collector/Nurse 4)*. Staff made sure to collect patient phone numbers whenever possible. They indicated to patients that they should call or visit at any signs of infection even if they did not have an appointment scheduled.

Based on these procedural changes, staff noticed increased patient satisfaction particularly due to increased outpatient follow up, which provided encouragement during implementation.

*“Calling the patients, has made me really happy. They really love to hear me call and say, ‘How are you feeling? Are you feeling okay?’ And that gives me some satisfaction.” (Data Collector/Nurse 5)*

Individual incentives played a smaller role in motivating change. Some participants reported that the checklist felt like a form of legal protection in surgery:

*“Legally you are insured, when you fill the checklist. If something has happened you have the record.” (Data Collector/Nurse 3)*

In order to facilitate acceptance of Clean Cut, staff leveraged existing relationships and one-on-one conversations with individuals who were initially resistant to changing practices. For instance, with a more consistent OR team, the entire team worked together to produce a positive team dynamic. Some teams made completing the checklist a fun activity by embracing the parts that at times felt redundant:

*“It's becoming fun. They are enjoying it. The introducing the name of the surgeons. Because already obviously they know us they don't want to ask repeatedly our names. But currently, even we have resident for the last four months, they speak out their names loud and clear names every time despite we know each other. It's kind of fun and important consideration as well.” (Surgeon 4)*

At other sites, a cohesive OR team was able to communicate mistakes made early on and confront early resistors. Regardless of existing team dynamics, one-on-one conversations were utilized to facilitate adoption at all sites. These conversations were led by Clean Cut leaders on site, country leads, clinical leads, and in some cases scrub nurses who were driving SSC use. At one of the larger sites, one participant noted that *“there was a little reluctant from our side, but scrub nurse insists in doing the checklist, especially in the emergency hours.” (Surgeon 3).* Overall, individual conversations were based on increasing understanding of the program in order to enhance commitment:

*“People give you results based on the level of commitment they have, and the level of commitment is always dependent on the level of understanding to the process. I think so long as you bring the issue on and discuss it and then convince people that it's important and it's very helpful for the patients and for us as well … By doing that, I think people want to be part of that culture change.” (General Practitioner 1)*

#### Knowledge, beliefs, and education

Across all study sites, it was essential to use an evidence-based approach when training hospital staff for the intervention, and to orient the entire OR staff to the program rather than just Clean Cut team members. The most effective driver of change was increasing the level of knowledge surrounding surgical site infection, which frequently required correcting previously held erroneous beliefs.

*“So, before Clean Cut, it was only one or two rooms who usually use the checklist, and even in those areas it was not daily use. Some physicians use it, some do not feel comfortable to use it. But after Clean Cut and after the renovated OR, they’re trained to use the checklist because we had this small session on checklist use with the OR nurses and anesthetists in addition to any other team that is involved in how to use it” (Surgeon 1)*

Study participants mentioned that it was not sufficient to only orient Clean Cut participants. Because the SSC and the intervention specifically required all OR personnel to be compliant with safety standards, there is a similar need to use the same evidence-based implementation approach across the hospital.

*“Even if we have to scale up to the other hospitals about this Clean Cut project, it is better if we initiate or introduce the whole OR staff or the OR team, at the beginning of the project about the objective and the issue, ...the data collector and also the coordinator.” (Data Collector/Nurse 6)*

#### Data collection

Not all sites produced new methods of data collection or modified what was provided by Clean Cut. However, at one of the larger sites where communication was difficult, the team created a logbook to track patients in a more comprehensive way than what had previously been done. The team periodically met to validate the data, check for errors, and complete missing patient data.

Financial incentives were a complicated issue that proved to be both a barrier to continued data collection and a facilitator. As one respondent noted, the extra work is facilitated by compensation due to the nature of low salaries: “*Scrub [techs] even surgeons are specifically low paid...so these incentives might be helpful to collect data which are accurate.” (Surgeon 3).*

### Permanent changes

At all four Clean Cut sites, study participants noted that the most important lasting change of implementation would be improvements in OR behaviors around infection prevention. In particular, each site had adopted use of the SSC in the OR, citing improved quality of patient care as a long term motivator. Another perioperative change that was consistently endorsed was increased use of autoclave internal sterility indicators. Once these changes were incorporated into the routine workflow of the operating room, they were perceived as permanent.

*“The changes will [be] permanent, the activities that we do in the OR like scrubbing, instrument processing, gown sterilizing, about using the indicator. These will be permanent.” (Data Collector/Nurse 7)*

While these process measure changes were thought to be permanent, there were mixed responses regarding data collection. Some participants thought data collection would continue but did not know of any specific plans. Others felt that continued data would be unnecessary if there was systemic change in behavior after Clean Cut. One site thought some data collection was important to continue on-site but not follow-up phone calls, which was likely a response to patients lost to follow-up.

Similarly, we heard mixed responses regarding antibiotic prophylaxis. Some hospitals reported increased vigilance around giving antibiotics in the OR, while some still had ongoing resistance to timing of administration at the time of the interview. At sites where antibiotic prophylaxis was not always given in the OR, the variability was seen mostly in emergency cases and obstetric cases where prior practice was to give antibiotics on the wards.

### Identified requirements for future implementation

Participants provided advice they would have for future sites, through which we identified several important strategies for future implementation of Clean Cut (Table 3). A lack of material resources was identified as a major barrier to success, and thus it was also identified as a high priority issue to address during Clean Cut implementation.

**Table 3:** Strategies for Future Implementation

Strategy	Participant Quote
Assess resource availability before intervention and engage hospital leadership	“The human power, adequate manpower, sterility indicator and the office with a printer and a computer. This will happen better if these things are fulfilled at the beginning or initially, about the project, so that, I think, it might go smoothly.” Data Collector/Nurse 6
Create a sense of responsibility throughout the hospital staff	“To continue the data collection, everybody in the hospital who’s involved in patient care should have a complete understanding of the prevention of surgical site infection. That way, if they perceive that they are expected or they owe it as a common goal, then it will continue.” Data Collector/Nurse 7
Provide training beyond specific Clean Cut implementers	“If we engage more people, if we disseminate the information for more people, I think it’s easy to have an improvement. For example, for the gown and drapes I told you we don’t use the inside indicators because, the people at the sterilization room are not involved in that meeting. So if we involve more people, it’s easy, I think.” Data Collector/Nurse 1
Incorporate data collection into daily routines	“Not extra but as part of our daily job’s. Integrate into other jobs. Like using the safety checklist or something.” Data Collector/Nurse 1

Another high priority issue was creating a sense of responsibility among all hospital staff involved in infection prevention standards to facilitate implementation. Participants articulated this in a myriad of ways, including increased education to make sure staff understood the importance of surgical infection reduction. Others suggested that creating overall awareness is enough to cultivate a common goal.

Similar to creating ownership, participants suggested that disseminating training information and data results beyond Clean Cut members, and that sharing the results with all individuals involved in surgical site infection prevention practices would allow for faster and more successful implementation. Increased training for nurses would also alleviate the issue of manpower that data collectors found to be a burden.

Another identified way to decrease the sense of job burden was to ensure that data collection was incorporated into daily routines. As previously noted, there was not previously a practice of accurate data collection at any of the participating sites. Integrating this practice into daily routine of all OR staff, beyond the smaller Clean Cut team, was thought to promote sustainability.

Two other suggestions are worth noting: one was to communicate with hospital leaders via email ahead of scheduled implementation; the other was that participants at TASH, a leading academic university, endorsed the strategy of leveraging training institutions because residents who experience Clean Cut there can relay that knowledge in their next position at a different hospital.

## Discussion

The aim of Clean Cut is to reduce surgical infections by promoting improved adherence with the SSC and strengthening the processes and teamwork essential for its completion. Our qualitative approach provided the opportunity to gain specific, in-depth feedback of Clean Cut from a diverse group of staff. We elucidated barriers and facilitators which varied across sites and participants but contained common themes. Key barriers included material resource limitations, feelings of job burden, existing gaps in infection prevention education, and communication errors during data collection. The common facilitators included strong hospital leadership support, motivations based on patient outcomes, and organized Clean Cut training sessions. Participants endorsed new procedures as permanent changes when they were incorporated into the daily OR routines, such as confirming sterility through internal sterility indicators.

The strategies we identified for future projects can be prioritized in three overarching implementation science constructs: available resources, process engagement, and characteristics of individuals involved in change, as described in the Consolidated Framework for Implementation Research (CFIR) [19].

An early key step in Clean Cut implementation was assessing available resources (e.g., sterility indicators, alcohol rub and running water) [20]. Particularly as described in CFIR, resources also include “physical space, and time” [19], which was evident in our study in the expression of job burden. While it is unrealistic to solve all resource gaps pre-implementation, success will be facilitated by stressing the minimum physical resources to complete the SSC and Clean Cut data collection. Particularly in the setting of LMICs, initial assessments of resources will be important as this may vary widely from site to site. Similarly, the organization should recognize the potential for staff to perceive the intervention as extra work and make guided efforts to incorporate data collection into daily routines. An initial assessment of these limitations is a critical aspect of baseline analysis of new sites in order to individualize Clean Cut to each hospital. [20] Further, we suggest that information regarding resource limitations and procedures should be shared with hospital leadership, given that these individuals can make tangible investments.

Outside of the OR, one specific resource issue was the availability of paper for data collection and communication between staff. The move to electronic data capture through the use of smart phones, which were nearly universally available, is an important advance for the data collection process and is currently being undertaken by the Ethiopian teams. This is an advancement that could be considered in other settings where data collection tools are not widely used.

Our study findings also emphasize the importance of engagement at all levels of hospital staff. In the first stages of Clean Cut implementation, each site underwent process mapping of procedures related to compliance with the six infection prevention standards outlined in Clean Cut [20]. Our qualitative findings suggest that the stakeholders who are identified during this process should be involved in presentation of baseline results and should remain involved in the 6 month intervention period. How key information is shared across organizations has been widely studied, and demonstrates that involving all stakeholders is key to success [19, 21]. Promoting communication is critical for ensuring that the staff identified are engaged and educated with evidence-based changes in order to facilitate sustainable improvements across relevant departments [21]. This should include those who are not directly involved in the OR, such as laundry room staff and ward nurses.

We found that a powerful theme emerged around a sense of shared responsibility within individual hospitals. These findings are largely consistent with prior literature in similar settings, as creating a sense of local ownership of the intervention at different levels of the hospital is a common theme across studies in Ethiopia [8, 22]. In contrast, confusion surrounding key objectives has been shown to be a barrier in quality improvement work [23]. Clean Cut aims to improve patient outcomes by reducing surgical infections, a major motivation for staff to engage in process changes. Peer-to-peer dissemination of outcomes information facilitated Clean Cut implementation and is a strong generator of overall enthusiasm broadly in healthcare interventions [19]. This pattern supports the theory that enthusiasm and understanding of the intervention drives behavior change, and that team-based approaches focusing on the how and why are important for success [7, 22].

Our study highlights the importance of leadership from diverse sources. In some cases, head surgeons championed the implementation, and in other cases, general practitioners and OR managers encouraged change. Occasionally, scrub nurses themselves promoted vocal SSC use and proper checks of sterility throughout OR procedure. While hierarchy is shown to be a major barrier in high income countries, we did not find this to be a driving force that determined success or failure in Clean Cut amongst our sites [7, 8]. Overcoming existing knowledge and beliefs inconsistent with Clean Cut was a larger barrier than overcoming hierarchy-based resistance. Improving training and promoting ownership throughout the entire institution was more impactful than a single champion.

### Limitations

Due to our purposeful sampling strategy, our recruitment was limited to individuals directly involved in Clean Cut, and thus our sample did not include other hospital staff not formally involved in the program, nor did we obtain perspectives of hospital staff who did not participate in Clean Cut training session. We were able to recruit 20 participants from a range of disciplines, and qualitative research methodology suggests this should be enough to reach theme saturation [10].

Further, participants may have been affected by response bias based on the interview structure, or the general desire to report what the interviewers were expecting. This was mitigated by neutrally phrased interview questions. Additionally, the responses were inherently influenced by the fact that all participants had been engaged in Clean Cut for several months by the time of the interview. We therefore missed initial impressions. However, with the additional experience, participants were able to reflect on longer-term implications. Our participants were purposefully limited to people who inherently had a minimum level of acceptance of the program and had a high level participation compared to other hospital staff so as to better understand their experiences in implementing Clean Cut; future studies should include a broader participant pool.

## Conclusion

The findings of this study underscore the importance of engaging hospital staff and involving hospital leadership from the beginning of implementation at future Clean Cut sites. We have highlighted major gaps as well as areas of success that are likely to be relevant at additional facilities. These areas that we have highlighted are not only relevant to Clean Cut, but underscore the impact of context specific factors especially relevant in LMICs where resource and cultural variation are essential to consider when implementing quality improvement programs. Further, the data dissemination through Clean Cut within each site and the lessons learned by team members contributes to information dissemination at a national level. Hospital providers in Ethiopia are frequently moved between hospitals, which has already shown to increase knowledge of Clean Cut, as past team members bring their experience to new positions. Lastly, Lifebox has shared detailed reports with the Federal Ministry of Health in Ethiopia. As awareness of Clean Cut and interest in expanding the program continues, these findings will contribute to successful implementation strategies.

## Additional file

Additional file 1: Clean Cut Qualitative Interview Guide. (DOCX 28 kb)

## Data Availability

The data analyzed during the current study are available from the corresponding author on reasonable request.

## Abbreviations

CFIR: Consolidated Framework for Implementation Research
IRR: Inter-rater reliability test
JUSH: Jimma University Specialized Hospital
LMIC: Low and Middle Income Countries
MII: Menelik II Referral Hospital
OR: Operating Room
SSC: WHO Surgical Safety Checklist
SSI: Surgical Site Infection
TASH: Tikur Anbessa Specialized Hospital
