# Supplementary material for: Qualitative outcomes of Clean Cut: implementation lessons from reducing surgical infections in Ethiopia
Source: BMC Health Serv Res. 2019 Aug 17;19:579. doi: 10.1186/s12913-019-4383-8 (PMC6698005; doi:10.1186/s12913-019-4383-8)
Supplement: Supplementary file 1 — Clean Cut Qualitative Interview Guide. (DOCX 28 kb) [file 12913_2019_4383_MOESM1_ESM.docx]

# additional file 1: Clean Cut Qualitative Interview Guide

| Participant ID: |  |
| --- | --- |
| Date: |  |
| Interviewer Name: |  |
| Project Site: |  |
| Participant Role  (in Clean Cut/Hospital): |  |
| Others present: |  |

## **Questions**

## Participant Information

1. What is your role in the hospital?
   1. What is your experience in that role?
2. How long have you worked in this hospital?
3. How many operations did you participate in last week?
   1. (Is that a normal amount for you?)
4. What is your role in the Clean Cut project?

## Pre-Implementation

1. Was the Surgical Safety Checklist used in your hospital before Clean Cut started?
   1. If so, how was the checklist being used?
   2. If so, who was responsible for reading the checklist in the operating room?
2. Before Clean Cut came to your hospital, how do you think your hospitals was doing with...
   1. Giving antibiotic prophylaxis within an hour of incision
   2. Using sterile gowns
   3. Using sterile drapes
   4. Using sterile gloves
   5. Performing gauze counts both before and after an operation
   6. Checking instrument sterility
   7. Washing or alcohol rubbing hands before surgery

## Perceptions of Baseline Data & Interventions

1. Did you participate in a meeting at your hospital where baseline data were presented about the Clean Cut project and patient infection rates?
2. Did anything surprise you from the presentation of Clean Cut baseline data?
   1. Why?
3. What changes has your hospital made based on the information presented?
   1. Giving antibiotic prophylaxis within an hour of incision
   2. Using sterile gowns
   3. Using sterile drapes
   4. Using sterile gloves
   5. Performing gauze counts both before and after an operation
   6. Checking instrument sterility
   7. Washing or alcohol rubbing hands before surgery
   8. How has this been a useful intervention or not?
   9. How did the surgical team accept or not accept this change?
4. Describe any resistance to change during implementation so far.
   1. Could these be/were they overcome, and if so how.

## General process

1. What was the biggest problem you faced in completing your work before the safe surgical checklist was implemented?
   1. What is the biggest problem you face in the OR today? Why?
2. Who helps perform the checklist / Who participates in the checklist procedure?
   1. How much support is there to use the surgical safety checklist during every surgery?
   2. Where does this support come from?
   3. Describe an area of your job where you could use more support.
3. Why do you use the surgical safety checklist?
4. What advice would you give other hospitals to prepare for implementing the SSC?
5. Have you seen or used any of the process maps created to identify gaps in each of the six Clean Cut infection prevention processes?
   1. Were these process maps useful? Did they help you identify areas that could be improved?
   2. Were there any challenges with using the process maps? How could they be improved?

## permanent changes

1. Does your hospital have any plans to collect data after Clean Cut is over?
   1. Why or why not?
   2. If yes, describe these plans.
2. Do you think the hospital will continue any of the recent interventions once the project ends, or not?
   1. Which interventions do you think will be permanent changes in your hospital? Why?

**Is there anything else that you’d like to tell me about the surgical safety checklist?**

**Is there anyone else you think I should talk to about using the checklist at your hospital?**
